# The mitochondrial genome of the long-billed plover, *Charadrius placidus* (Charadriiformes: Charadriidae)

**DOI:** 10.1080/23802359.2017.1292473

**Published:** 2017-02-23

**Authors:** Mu-Yeong Lee, Hey Sook Jeon, Sang-Hwa Lee, Junghwa An

**Affiliations:** a Animal Resources Division, National Institute of Biological Resources, Incheon, Republic of Korea;; b Graduate Program in Cellular Biology and Genetics, College of Medicine, Chungbuk National University, Cheongju, Republic of Korea

**Keywords:** *Charadrius placidus*, mitochondrial genome, long-billed plover

## Abstract

The present study provides the first full mitochondrial genome sequence of a long-billed plover from South Korea. This mitogenome is 16,895 bp in length and consists of 13 protein-coding genes, 2 ribosomal-RNA genes, 22 transfer-RNA genes, and a non-coding control region. Overall, base composition was: A, 31.4%; C, 31.1%; G, 13.8%; and T, 23.8%. A frameshift mutation in the *ND3* gene was identified and a tandem repeat (AACA) was observed in the D-loop region. The phylogenetic analysis based on concatenated coding genes indicated Charadriidae’s monophyly. These results contribute to further understanding of long-billed plover phylogenetic relationships and species identification.

The long-billed plover, *Charadrius placidus*, is a small–medium wading bird in the family Charadriidae. It breeds in western, northern, central, and north-east China, North Korea, and Japan and spends winter in Nepal, north-east India, southern China, Taiwan, and the Korean Peninsula (Hayman et al. 1986). Breeding *C. placidus* was recently reported in South Korea (Kim et al. 2002). Its populations are considered to be decreasing due to habitat destruction, such as that resulting from gravel banks in South Korea (Kim et al. 2009; BirdLife International 2016). Although *C. placidus* is currently classified as an endangered species II by the Ministry of Environment of Korea, it is listed as Least Concern on the International Union for Conservation of Nature red list. To the best of our knowledge, the mitochondrial genome of the genus *Charadrius* was not available and, therefore, in the present study, we sequenced and characterized the complete mitochondrial genome of *C. placidus*, enriching the basic genetic information on this species and on this genus.

A specimen of *C. placidus* (IN671) was collected from the Taehwa River, Wolsan-si, Kyeongsangnam-do, South Korea, and deposited in the National Institute of Biological Resources (NIBR) at Incheon, South Korea. Total genomic DNA was extracted from the muscle tissue using the DNeasy Blood & Tissue Kit (Qiagen, Valencia, CA) following the manufacturer’s protocol. The mitochondrial genome of *C. placidus* was amplified and sequenced using 16 pairs of primers, and the sequences obtained were assembled, edited, and checked in Geneious Pro 8.1.9 (Biomatters Ltd, Auckland, New Zealand; Kearse et al. 2012). The mitogenome sequence of *C. placidus* was deposited in GenBank under accession number KY419888. This circular mitogenome was 16,898 bp in length and comprised 13 protein-coding genes, 22 transfer-RNA (tRNA) genes, 2 ribosomal-RNA genes, and 1 control region (D-loop region). Its overall base composition was 31.4% A, 31.1% C, 13.8% G, and 23.8% T, thus having a slightly higher A+T percentage (55.2%). Except for the *ND6*-subunit gene and eight tRNAs, all genes were encoded on the H-strand of the mitogenome, as in other vertebrates. Tandem four-base repeats (AACA) were found in the D-loop region (1315 bp), which was located between tRNA-Glu and tRNA-Phe. The *ND3* gene had a frameshift ‘C’ mutation at position 174, which has also been reported in some birds and turtles (Mindell et al. 1998). This mutation was also identified in all the species used in the phylogenetic analysis, except *Vanellus cinereus* (accession KM873665).

The phylogenetic analysis performed in MEGA 6 (Tamura et al. 2013) based on the concatenated sequences of the 13 protein-coding genes (11,401 bp) and using the neighbor-joining method (Figure 1) showed the monophyly of Charadriidae. The results of the present study contribute to further understanding of the phylogenetic relationships within the genus *Charadrius* and provide valuable resources for molecular identification of species.

**Figure 1.**
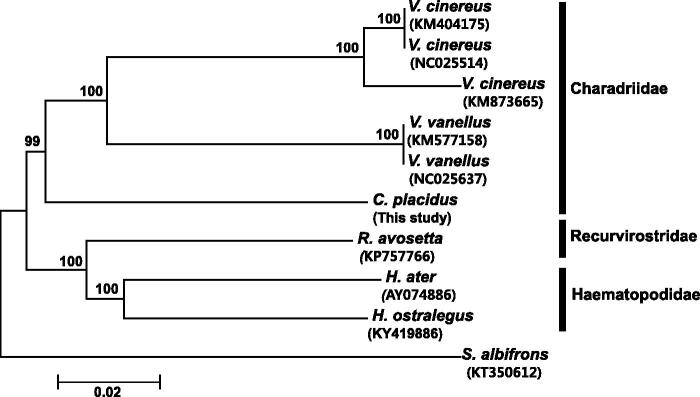
Neighbor-joining phylogenetic tree of the long-billed plover and six other species belonging to the families Haematopodidae, Recurvirostridae, and Charadriidae, based on the concatenated nucleotide sequences of 13 protein-coding genes. Numbers on nodes indicate bootstrap support value, based on 1000 replicates, and numbers below species names indicate their GenBank accession code.
